# Antibodies against invasive phenotype-specific antigens increase *Mycobacterium avium* subspecies *paratuberculosis* translocation across a polarized epithelial cell model and enhance killing by bovine macrophages

**DOI:** 10.3389/fcimb.2015.00058

**Published:** 2015-08-07

**Authors:** Jamie L. Everman, Luiz E. Bermudez

**Affiliations:** ^1^Department of Microbiology, College of Science, Oregon State UniversityCorvallis, OR, USA; ^2^Department of Biomedical Sciences, College of Veterinary Medicine, Oregon State UniversityCorvallis, OR, USA

**Keywords:** *M. avium paratuberculosis*, invasion, intestinal tract, antibodies, macrophage killing

## Abstract

Johne's disease, caused by *Mycobacterium avium* subspecies *paratuberculosis* (MAP), is a severe chronic enteritis which affects large populations of ruminants globally. Prevention strategies to combat the spread of Johne's disease among cattle herds involve adhering to strict calving practices to ensure young susceptible animals do not come in contact with MAP-contaminated colostrum, milk, or fecal material. Unfortunately, the current vaccination options available are associated with high cost and suboptimal efficacy. To more successfully combat the spread of Johne's disease to young calves, an efficient method of protection is needed. In this study, we examined passive immunization as a mode of introducing protective antibodies against MAP to prevent the passage of the bacterium to young animals via colostrum and milk. Utilizing the infectious MAP phenotype developed after bacterial exposure to milk, we demonstrate that *in vitro* opsonization with serum from Johne's-positive cattle results in enhanced translocation across a bovine MDBK polarized epithelial cell monolayer. Furthermore, immune serum opsonization of MAP results in a rapid host cell-mediated killing by bovine macrophages in an oxidative-, nitrosative-, and extracellular DNA trap-independent manner. This study illustrates that antibody opsonization of MAP expressing an infectious phenotype leads to the killing of the bacterium during the initial stage of macrophage infection.

## Introduction

Johne's disease is a chronic enteritis caused by the pathogen *Mycobacterium avium* subspecies *paratuberculosis* (MAP). The global burden of the disease is widespread and outdated studies suggest that the disease results in an economic loss of $250 million to $1.5 billion per year in culled herds and loss of milk production within the US dairy industry alone (Stabel, 1998; Ott et al., 1999). The most successful of current prevention strategies involves managing the spread of disease by implementing carefully planned calving practices to ensure that young animals receive colostrum and milk from Johne's-free dams. These practices prevent the exposure of young susceptible animals to contaminated feces, decrease the rate at which animals are culled and removed from the herd after testing positive for the bacterium.

Multiple vaccine formulations exist, though only one is commercially available in the United States. Overall, vaccination rates are generally low and herd-management is the most common and economically feasible form of Johne's prevention worldwide. Published studies, and the product information for the commercially available vaccine Mycopar (Boehringer Ingelheim Vetmedica, Inc.) explain that while vaccination limits the progression of cases to the clinical stage of the disease, it does not prevent shedding of MAP in the feces, nor does it prevent vaccinated animals from becoming infected (Wentink et al., 1994). Due to these factors and its associated cost, strict timeline of administration, and suboptimal efficacy, there is a continuous push to develop more efficacious vaccines to combat MAP infection. Unfortunately, the results obtained from the pipeline of determining host toxicity and vaccine efficacy from *in vitro* cultures and mouse models did not translate in a successful vaccine trial in ruminant hosts due to unappreciated differences in immunity and pathogenesis of the infection between animal species (Hines et al., 2014). Furthermore, the phenotypic changes that occur within MAP during infection (Everman et al., 2015) or during exposure to different environmental or host reservoirs (Cirillo et al., 1997; Patel et al., 2006; Alonso-Hearn et al., 2010) may result in ineffective vaccine efficacy. It is possible that due to the incorrect focus of vaccine development, chosen vaccine candidates are not representative of the most relevant antigens during the stages of Johne's disease in the animal. This is certainly a limitation of the current vaccine target approach, with consequent inefficient protection over the full course of the disease.

Compared to vaccine-induced (active) immunity, which requires the host immune system to mount a response to introduced antigens, passive immunity provides immediate protection in the form of pre-formed antibodies. Neonatal calves have a narrow repertoire of gammaglobulins due to their immature immune systems and early protection of the animal is provided by uptake of maternal immunoglobulins concentrated in the colostrum during the first feedings in the early hours of life. These colostrum-delivered antibodies provide immediate immunity against naturally occurring enteric and respiratory pathogens which can lead to deadly diarrheal and pneumonic diseases in animals that do not receive proper feedings of colostrum (Godden, 2008). Experimental vaccination of pregnant cows has shown to provide protection against pathogens such as *Escherichia coli* (Reiter and Brock, 1975; Nagy, 1980), *Cryptosporidium parvum* (Perryman et al., 1999), and rotavirus (Saif et al., 1983), by the resulting mounted antibody titers which are passed to the neonate during initial feedings of colostrum. This passive transfer of opsonizing antibodies enables host phagocytes to eliminate potentially harmful pathogens by phagocytosis and intracellular killing, or by triggering antibody-dependent cell-mediated cytotoxicity (ADCC) for the elimination of the pathogen in the mucosal tissues of young animals.

Previous studies have shown that MAP can reside within and acquire an infectious phenotype in the presence of milk, and in the mammary gland, with significant alteration in the gene expression of the pathogen (Koenig et al., 1993; Patel et al., 2006; Antognoli et al., 2008; Alonso-Hearn et al., 2010). This infectious phenotype may provide a novel and unstudied array of surface antigen biomarkers that may be used for the development of more effective preventative strategies. Considering the inherent susceptibility of young animals to infection, the altered and infectious MAP phenotypes from milk exposure, the protective mechanisms of passively obtained immunoglobulins, and the knowledge that young calves are at the highest risk of acquiring infection from uptake of contaminated milk, we hypothesize that maternal passive immunity may serve as a protective mechanism against the passage of MAP to young animals.

In the current study, we investigate the role of serum opsonization and its effect on the bacterial interaction with bovine epithelial cells and macrophages during infection. We reveal that serum opsonization of the infectious phenotype of MAP enhances the translocation of the bacteria across an epithelial barrier. Upon interaction with bovine macrophages, the immune serum opsonized pathogen expressing the milk-exposed infectious phenotype is rapidly killed. Based on our findings, we begin to understand how the method of passive immunity delivered with the adult cows' milk may serve as a successful approach for protection against acquiring MAP in the milk during the feeding of young animals.

## Methods and materials

### Bacterial culture

*M. avium* subspecies *paratuberculosis* strain K10 (ATCC BAA-968) was cultured at 37°C on 7H10 agar (BD; Franklin Lakes, NJ) supplemented with casein hydrolysate (1 g/L; BD), 10% (vol/vol) oleic acid, albumin, dextrose, and catalase (OADC; Hardy Diagnostics; Santa Maria, CA), and ferric mycobactin J (2 mg/L; Allied Monitor, Fayette, MO) for 3–4 weeks. Prior to experiments, a bacterial suspension was made in HBSS (Corning; Corning, NY), passed through a 22-gauge needle five times to disperse clumps, and allowed to settle for 10 min. The top half of the inoculum was used as a single-cell suspension for experiments (Patel et al., 2006).

### Mammalian cell culture

Madin–Darby Bovine Kidney (MDBK) epithelial cells (CCL-22) and murine RAW 264.7 macrophage cultures (TIB-71) were obtained from the American Type Culture Collection (ATCC; Manassas, VA). Both cells lines were cultivated in Dulbecco's Modified Eagle's Medium (DMEM) supplemented with 10% heat-inactivated fetal bovine serum (FBS; Gemini Bio-Products; West Sacramento, CA), at 37°C in 5% CO_2_. The SV40 mutagenized bovine macrophage cell line (BOMAC) was a gift from the USDA and cultivated in RPMI-1640 (Corning) supplemented with 10% heat-inactivated fetal bovine serum at 37°C in 5% CO_2_ (Stabel and Stabel, 1995).

### Serum samples

*Bovine Serum*. Serum samples that were positive and negative for antibodies against MAP antigens, as determined by USDA diagnostic ELISA and Western blot against whole cell MAP lysates, were obtained from the USDA Sample Repository (Ames, IA) (Table 1). Serum samples from 10 positive and 10 negative animals were acquired, positive and negative samples were pooled, and aliquots stored at -80°C prior to use. *Mouse serum*. Six-week old female C57BL/6 mice were purchased from Jackson Laboratory (Bar Harbor, ME) and held under observation for 1 week prior to use. MAP was exposed to milk as described below and whole MAP lysate was isolated by bead-beating bacteria with 0.1 mm glass beads in HBSS 5 times for 30 s in a Mini-BeadBeater (Biospec Products; Bartlesville, OK) at a speed of 4800 oscillations/min. Protein lysate was cleared of intact cells and cell wall debris by centrifugation at 9500 × g for 30 min at 4°C and plated to confirm no viable bacteria existed in the antigen preparation. Ten mice were pre-bled for control non-immune serum prior to immunization on day 0. Animals were injected subcutaneously with 0.1 mg of MAP antigen in incomplete Freund's adjuvant (Sigma; St. Louis, MO) in three dorsal administration sites on day 1 and with 50 μg of MAP antigen in incomplete Freund's adjuvant in two dorsal sites every 2 weeks. Test-bleeds were conducted to confirm antibody titer by Western blot analysis using a 1:5000 dilution of serum as the primary antibody and detected using a goat anti-mouse IRdye800 secondary detection antibody as per manufacturer's instructions (Licor; Lincoln, NE). Blood was collected via cardiac puncture and serum was isolated by centrifugation at 5500 × g for 15 min at 4°C. Samples were pooled and aliquots stored at −80°C. All animal procedures were completed in strict accordance with guidelines set by the institutional animal care and use committee (Oregon State University Animal Care and Use Protocol #4490).

**Table 1:** **Serum samples and MAP detection via PCR, agar growth, and ELISA tests**.

**Accession number**	**Date collected**	**Shedding status**	**Direct PCR**	**Solid media (Avg CFU/Tube)**	**Parachek ELISA**
**NEGATIVES**					
09-029155	11/9/2009	Negative	Negative	Negative	0.07
09-029155	11/9/2009	Negative	Negative	Negative	0.057
10-007776	2/17/2010	Negative	Negative	Negative	0.08
10-013528	3/19/2010	Negative	Negative	Negative	0.0535
10-013528	3/19/2010	Negative	Negative	Negative	0.0545
10-013528	3/19/2010	Negative	Negative	Negative	0.0735
10-013528	3/19/2010	Negative	Negative	Negative	0.0695
10-007776	2/17/2010	Negative	Negative	Negative	0.08
10-044414	9/30/2010	Negative	Negative	Negative	−0.021
10-044414	9/30/2010	Negative	Negative	ND	−0.024
**POSITIVES**					
NA	2/13/2007	High	ND	TNTC	3.095
NA	6/20/2007	High	ND	TNTC	2.439
548443	Apr-08	Low	30.7	6	1.03
547344	Apr-08	High	20.1	TNTC	2.2175
547344	Apr-08	High	23.8	TNTC	1.6765
09-012862	Aug-09	High	30.0	300	1.311
10-036633	Jul-10	High	22	4900	1.84
11-056306	11/7/2011	High	27.5	317.5	2.176
12-003868	2/1/2012	High	28.3	517.5	2.965
12-005807	2/15/2012	High	28.9	8500	1.511

### Bacterial exposure to raw milk and serum opsonization

Freshly collected whole milk from Holstein-Friesian cows was collected from a bulk tank at the Oregon State University Dairy Center. Milk was separated into three fractions by centrifugation for 20 min at 13,000 × g; milk fat (top layer) and milk particulate (pellet) were discarded and raw milk (middle fraction) was treated with polymyxin B (5 μg/ml), amphotericin (22 μg/ml), carbenicillin (25 μg/ml), and trimethoprim (2.5 μg/ml) overnight at 4°C. Samples were centrifuged again for 20 min at 13,000 × g to pellet any precipitated material, supernatant was collected, and aliquots frozen at −20°C. Prepared raw milk samples were inoculated with MAP for 24 h at 37°C with shaking at 200 rpm. To isolate milk-exposed MAP, suspension was centrifuged at 2000 × g for 15 min at 4°C, and pellet was washed in HBSS twice for 10 min at 3500 × g at 4°C. For *in vitro* opsonization experiments, 1.5 × 10^7^ milk-exposed MAP were mixed with a 1:50 dilution of serum in 1 ml total in HBSS and incubated for 1 h in a rotating hybridization oven at 37°C. Mock controls were incubated in an identical manner in the absence of serum.

### Invasion assays

MDBK, BOMAC, or RAW 264.7 cells were seeded into 48-well plates and grown to 80% confluence prior to experiments. Cells were infected at an MOI of 10:1 with opsonized MAP samples and controls in complete DMEM or RPMI and infections were synchronized at 220 × g for 5 min prior to incubation at 37°C with 5% CO_2_. For invasion assays, infections were incubated for 15, 30, 45 min or 1 h depending on experimental design, cells were washed three times with HBSS and depending on experiment, fresh DMEM supplemented with amikacin (200 μg/ml) was added to each well for 2 h to kill extracellular bacteria and washed 3 times with HBSS prior to lysis (Bermudez and Young, 1994). To quantify bacterial uptake cells were lysed with 0.1% triton X-100 in deionized water, samples collected, serially diluted, and plated for CFU determination.

### Transwell monolayer translocation assays

A transwell insert (Costar; Tewksbury, MA) with 3.0 mm pores was inserted into a 24-well tissue culture plate (Corning). 10^4^ MDBK cells/well were added to the apical chamber of each well and DMEM was supplemented into the basal chamber upon seeding. Both apical and basal chamber media was replenished with fresh media every other day. The integrity of the monolayers was measured every 2 days by two different methods: Trypan Blue permeability and transmembrane resistance. Every 2 days, 0.4% trypan blue was added to the apical chamber of test wells and basal chamber samples were collected at 1, 5, 10, and 30 min. Samples were measured for trypan blue absorbance at 580 nm using a spectrophotometer (Boiadjieva et al., 1984; Mangum et al., 1990). Transwell cultures were considered intact monolayers and used for assays once the trypan blue permeability read-out was less than OD_580_ 0.010 at 30 min post-exposure and transmembrane resistance was greater than 400 Ω/cm^3^ (Bermudez et al., 2002). For translocation assay, MAP was exposed to milk and opsonized as described above. Transwell inserts were infected with 10^6^ MAP/well in the apical chamber in DMEM and fresh media was added to the basal chamber and incubated at 37°C in 5% CO_2_. Samples were obtained at 6- and 24-h post-infection by collecting 500 μl from the basal chamber, at which time media was replaced with equal volume media which was collected at 24 h post-infection. Data from 6-h samples were obtained from direct quantification of colonies and 24-h samples were obtained by the addition of samples quantified from both the 6- and 24-h timepoints.

### Mammalian cell response inhibitor assays

To block cellular response mechanisms, cells were treated with 10 U/ml catalase (Sigma), 300 U/ml superoxide dismutase (Sigma), a combination of 10 U/ml catalase and 300 U/ml superoxide dismutase, 50 μM diphenyleneiodonium chloride (DPI; Sigma), 250 μM *N*^*G*^-monomethyl-l-arginine (L-NMMA monoacetate; ENZO Life Sciences; Farmingdale, NY), DNase I 100 U/ml (Roche; Basel, Switzerland) and their respective buffers of 0.1 M potassium phosphate (catalase and superoxide dismutase separately), 0.2 M potassium phosphate (mix of catalase/superoxide dismutase), dimethylsulfoxide (DMSO; Sigma; used for DPI buffer), deionized water (L-NMMA), and DNase Buffer alone (DNase) as previously described (De Assis et al., 2000; Brinkmann et al., 2004; Aulik et al., 2012). Host cells were incubated for 30 min with each compound or buffer in RPMI and washed two times with HBSS prior to bacterial infection. Cells were then infected as described above with opsonization samples suspended in RPMI supplemented with each inhibitor or buffer for 15 min, washed, lysed with 0.1% triton X-100 in deionized water, serially diluted, and plated for CFU. Milk-exposed opsonized MAP samples and host cells were incubated in cellular inhibitors individually for the same amount of time to determine the effect of each inhibitor on bacterial viability and host cell viability and quantified using CFU counts or trypan blue staining (0.4% w/v), respectively.

### Statistical analysis and data interpretation

Results are reported as the mean of at least 2 replicate experiments each performed in triplicate ± standard error. Statistical comparisons between experimental groups and control groups were determined using the Student's *t*-test with *p* < 0.05 denoting statistical significance. GraphPad Prism version 6.0 software was used for the construction of graphs, data interpretation, and statistical analysis. The data presented in this manuscript is determined by the CFU of MAP recovered from the intracellular or extracellular environment (dependent on the experiment) divided by the original CFU amount of bacteria added to each well prior to infection. This value is then multiplied by 100 to reach the percent uptake/survival/recovery as reported in the graphs. To clarify the data, 4 vs. 6% uptake means that 4% of the original inoculum was recovered as intracellular CFU data compared to 6% of the inoculum of the second sample. We present our data in this manner to normalize for the variation in inocula within each well, sample, and treatment type. However, we do ensure that the original inocula are relatively close, making sure the opsonization or chemical treatment of the bacteria isn't affecting the initial infecting inocula prior to experiments (data not shown).

## Results

### Whole serum opsonization influences the interaction of MAP with MDBK epithelial cells

**Figure 1:**
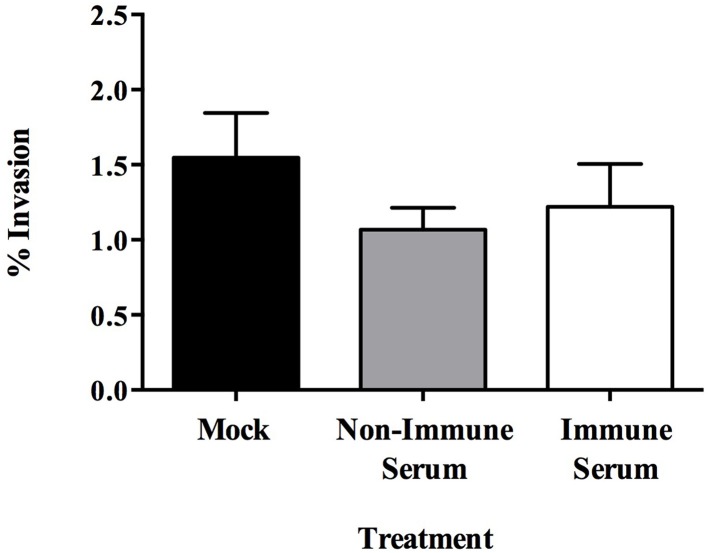
**Effect of opsonization of milk-exposed MAP on invasion of MDBK epithelial cells**. MAP incubated in raw milk for 24 h was isolated and opsonized in PBS (mock), or 2% serum from uninfected (non-immune), or infected (immune) cattle for 1 h at 37°C. MDBK monolayers were infected for 1 h at 37°C before collection of cell lysate. Percent uptake was calculated by (amount of intracellular bacteria/amount of bacteria added to the well) × 100. Data represents mean ± SEM of two independent experiments each performed in triplicate. Differences not significant (*p* > 0.05) as determined by a Student's *t*-test.

Opsonization aids in the uptake of pathogens by host phagocytes and has been demonstrated to enhance killing of intracellular pathogens during infection (Weber et al., 2014). Previous investigations have shown that opsonization of plate-grown MAP increases the efficiency at which the bacterium is able to be ingested and increases survival within the intracellular compartment (Hostetter et al., 2005). However, the effect opsonization has on the uptake of MAP by the intestinal mucosa is unknown. To determine the effect of opsonization on the interaction between MAP cultured in milk (hereafter referred to as the infectious phenotype) and the bovine intestinal epithelium, we evaluated how opsonization influenced the invasion of and the translocation across polarized bovine MDBK epithelial cells. The infectious phenotype of MAP was recovered and opsonized with PBS (mock), pooled bovine serum from Johne's negative animals (non-immune serum), or pooled bovine serum from Johne's positive animals (immune serum) (Table 1). Invasion assays indicated that neither non-immune nor immune serum opsonization had any significant effect on the ability of MAP to invade MDBK epithelial cells compared to mock opsonized samples (Figure 1). While the epithelial cell invasion capability of MAP isn't significantly affected by opsonization, the mechanism of uptake may provide a difference in the survival or efficiency of translocation across the epithelium.

**Figure 2:**
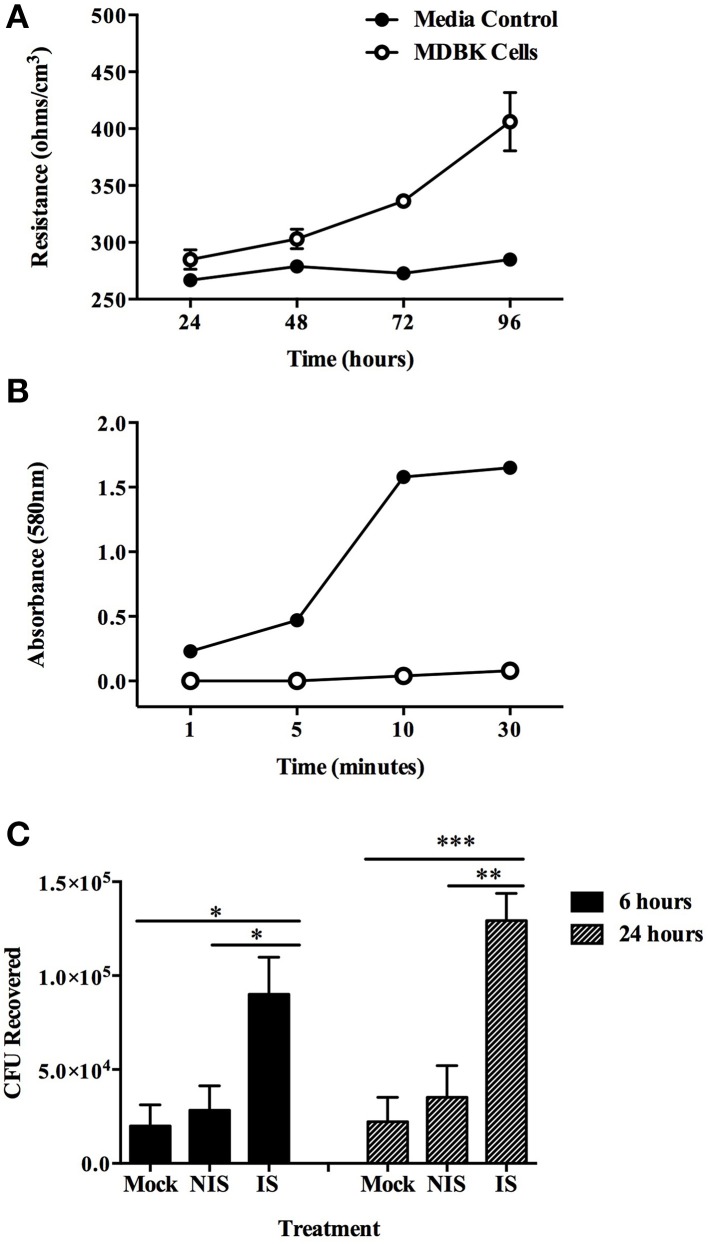
**Translocation of opsonized milk-exposed MAP across an MDBK epithelial transwell monolayer**. Apical chambers were seeded with 10^4^ MDBK cells and monolayer integrity was measured. Trypan blue exclusion assay was measured every other day **(A)**, and transmembrane resistance of each well was measured every day until monolayer resistance reached 400 Ω/cm^3^ **(B)**. MAP was incubated in milk for 24 h and then opsonized with PBS (mock), serum from Johne's negative cattle (non-immune serum), or pooled serum from Johne's positive cattle (immune serum). The apical chamber of each transwell was infected with 10^6^ MAP and samples from the basal chamber were collected and quantified for translocated bacteria at 6 and 24 h post-infection **(C)**. Trypan blue permeability data represent mean ± SEM of three independent experiments each performed in duplicate; transmembrane resistance data represent mean ± SEM of eight wells and is representative of three independent experiments; translocation data represent the mean ± SEM of two independent experiments each performed in triplicate (^*^p < 0.05; ^**^p < 0.01; ^***^p < 0.001 as determined by a Student's *t*-test).

To investigate the fate of opsonized MAP after uptake by MDBK epithelial cells, a transwell culture assay was employed. After 4 days in culture, monolayers were determined to be impermeable and intact as demonstrated by both transmembrane resistance measurements (Figure 2A) and a trypan blue permeability assay performed 4 days after cell seeding (Figure 2B). Opsonization with immune serum resulted in enhanced translocation of MAP across an intact polarized MDBK epithelial cell monolayer with 3.1-fold more bacteria being recovered from the basolateral chamber after 6 h, and a 3.7-fold increase in recovered MAP after 24 h post-infection compared to non-immune serum opsonized bacteria (Figure 2C). These data indicate that opsonization with sera from Johne's infected, immune positive cattle has little effect on uptake of bacteria by MDBK epithelial cells, but results in significantly enhanced translocation of infectious MAP across the polarized epithelial cell monolayer during infection.

### Bovine macrophage uptake of serum opsonized MAP

**Figure 3:**
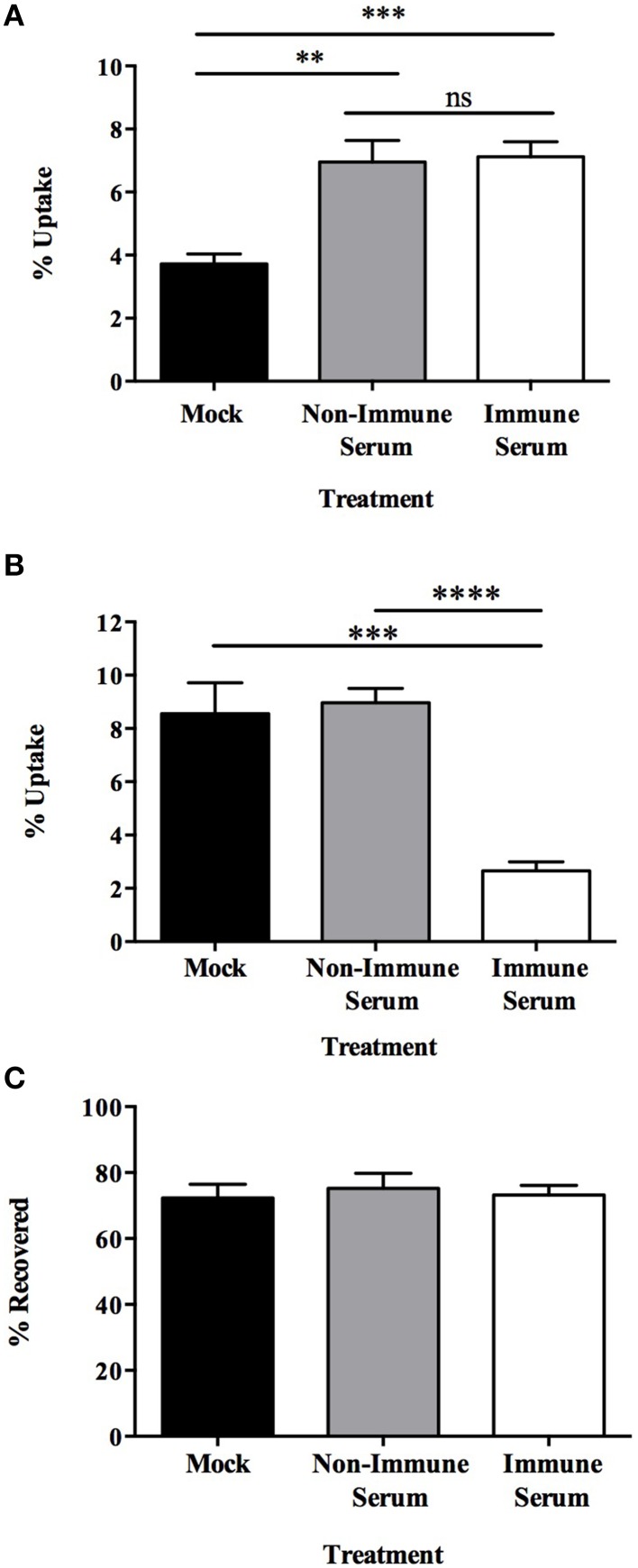
**Immune serum opsonization of milk-exposed MAP initiates phenotype which decreases uptake of bacteria by BOMAC via cell-mediated killing**. MAP were incubated in 7H9 broth **(A)** or milk **(B,C)** for 24 h, then opsonized with non-immune serum, immune serum, or PBS (mock) and used to infect BOMAC cells for 15 min. Samples were immediately collected and cell lysates **(A,B)** and supernatants **(C)** from each well were quantified for bacterial uptake and supernatant bacterial viability, respectively. Data shown represents the mean ± SEM of four independent experiments each performed in triplicate (^**^p < 0.01, ^***^p < 0.001; ^****^p < 0.0001 as determined by a Student's *t*-test).

Once established that opsonization of MAP increased the level of translocation across a polarized MDBK epithelial cell monolayer, we wanted to address whether enhanced translocation contributes to the virulence of MAP by allowing greater numbers to be taken up by and survive within tissue macrophages, or whether greater translocation results in enhanced uptake and subsequent killing of MAP by macrophages. To assess the MAP-macrophage interaction in this system, we analyzed what effect opsonization had on the uptake of MAP by an immortalized bovine macrophage cell line (BOMAC). Preliminary assays were carried out to determine if our experimental model was able to replicate the effect of macrophage uptake of MAP grown under standard conditions as previously described (Hostetter et al., 2005). Consistent with previous studies, opsonization of broth cultured MAP with serum samples results in a two-fold increase in uptake of MAP by bovine macrophages after a 15 min infection (Figure 3A). It was also shown that neither mock (PBS), non-immune serum, nor immune serum opsonization treatments of milk-cultured or 7H9-cultured MAP had any detrimental effect on the viability of each pool of bacteria prior to infection (data not shown), suggesting that any observable change is due to the response of the host cell and not due to a difference of the initial inoculum levels or from an impact on viability from serum components, culture, or opsonization treatments occurring before infection.

Prior studies demonstrated the increased ability of the infectious phenotype of MAP to invade MDBK epithelial cells (Patel et al., 2006), and our data indicates that the infectious phenotype also has a significantly greater rate of uptake by BOMAC cells after a 15 min infection as 8.55 ± 1.15% of MAP expressing the infectious phenotype are ingested by BOMAC cells (Figure 3B; black bar) compared to 3.72 ± 0.32% of 7H9-exposed MAP (Figure 3A; black bar) after infection (*p* = 0.0054). To determine the effect of the infectious phenotype on the interaction between opsonized MAP and BOMAC cells, infection assays were carried out to assess the amount bacteria that were able to be taken up by macrophages. Intracellular bacterial quantification demonstrated that the infectious phenotype resulting from milk exposure results in a 75% decrease in BOMAC uptake of immune serum opsonized samples compared to mock or non-immune serum opsonized MAP populations (Figure 3B). This decrease was observed over a variety of infection lengths and sample treatments. Initial 3 h assays, which included a 1 h infection followed by 2 h of antibiotic treatment, and short-term infections of 15 min, 30 min and 1 h in the absence of antibiotic treatment were carried out and result in nearly identical data (15 min assay shown in Figure 3B; other data not shown). Collectively, these data indicate that immune serum opsonization of infectious MAP results in a significant decrease in the amount of bacteria taken up by BOMAC cell cultures during infection over both long- and short-term infections.

Immunoglobulins in the serum, particularly IgG and IgA, are responsible for opsonizing pathogens and toxins to enhance uptake and subsequent killing, or for neutralization and protection from disease, respectively (Janeway et al., 2001). To ascertain which function the serum antibodies have in our model and the mechanism responsible for the decrease in uptake of immune serum opsonized MAP, the supernatant from each experimental group during BOMAC cell infection was collected and the number of viable bacteria quantified (Figure 3C). After 15 min of infection approximately equal numbers of MAP were recovered from the supernatant of mock, non-immune, and immune serum opsonized infections. These findings indicate that the decrease in uptake of MAP opsonized with immune serum was not due to a neutralizing effect of the antibodies blocking uptake by macrophages. Rather, the infectious phenotype opsonized with immune serum results in the killing of MAP as there is no difference in the viable amount of bacteria within the supernatants of each treatment. The mechanism used to kill immune serum opsonized bacteria is rapid, as significant changes in viability occur after only a short 15 min incubation period. In total, these data illustrate that this infectious phenotype of MAP serves as a key mechanism for recognition by immune serum and for the specific response initiated by BOMAC cells during infection.

### Determining macrophage mechanisms for killing of immune serum opsonized MAP

**Figure 4:**
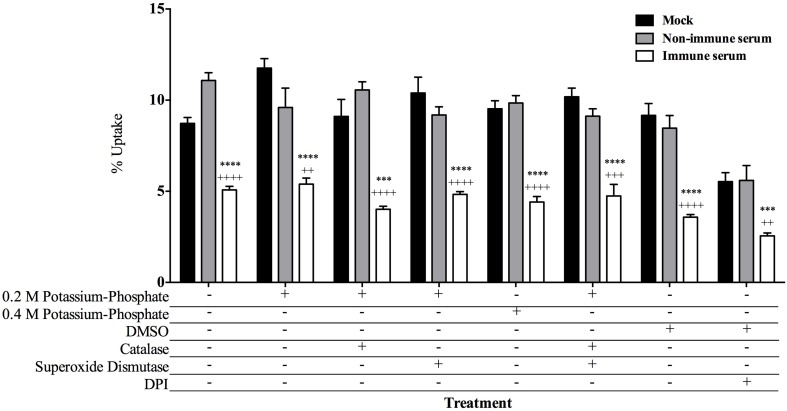
**Inhibition of cellular killing mechanisms and effect on uptake of opsonized MAP by BOMAC cells**. BOMAC cultures were pre-incubated in RPMI with the cellular mechanism inhibitors or respective vehicle controls for 30 min prior to infection as follows: catalase (10 U/ml)/0.2 M potassium phosphate, superoxide dismutase (SOD; 300 U/ml)/0.2 M potassium phosphate, diphenylene iodonium chloride (DPI; 50 μM)/DMSO, a combination of catalase/SOD (10 and 300 U/ml, respectively)/0.4 M potassium phosphate buffer. Each well was infected with 10^7^ mock, or serum opsonized milk-exposed MAP samples in RPMI in the presence of each respective inhibitor/buffer combination or buffer alone. After 15 min incubation, samples were washed, lysed, and quantified. Data shown represent mean ± SEM of two independent experiments each performed in triplicate (Mock treated samples compared to immune serum treated samples ^****^p < 0.0001, ^***^p < 0.001; Non-immune serum sample treatment compared to immune serum treatment samples ^++++^*p* < 0.0001, ^+++^*p* < 0.001, ^++^*p* < 0.01 as determined by Student's *t*-test).

Innate cellular mechanisms are vital for allowing host cells to react in a rapid and non-specific manner to pathogen associated molecular patterns (PAMPs) (Medzhitov, 2001), as well as antibody coated particles (Greenberg and Grinstein, 2002). The phagocytic vacuole is hard-wired to fuse with lysosomes and deliver numerous oxidative stress molecules that eliminate the pathogen within the vacuole (Janeway et al., 2001). To identify which host cellular mechanism(s) are being employed in the early killing of immune serum opsonized MAP, we inhibited a variety of cellular mechanisms known to be utilized during the process of phagocytosis. Though mycobacteria are well described to inhibit this fusion process (Kuehnel et al., 2001; Vergne et al., 2004), antibody-mediated phagocytosis may utilize different vacuole trafficking signals and fusion mechanisms which lead to a different outcome of mycobacterial survival. Chemical inhibitors of classical oxidative mechanisms were added to infections to determine if there was an effect on the viability of opsonized and non-opsonized milk-exposed MAP (Figure 4) over the course of the 15 min infection. The inhibition of hydrogen peroxide production by the addition of catalase, and the blockage of superoxide anion radical efficacy by superoxide dismutase (SOD) results in no change in the recovery of immune serum opsonized populations of MAP. As the conversion of superoxide by superoxide dismutase results in the release of oxygen and hydrogen peroxide, to combat both oxidative mechanisms we treated cells with a combination of catalase and superoxide dismutase in tandem, which results in no change from the control groups (Figure 4). The membrane bound NADPH oxidase enzyme complex is responsible for the production of a variety of reactive oxygen species (ROS) including but not limited to peroxides, oxygen radicals, and oxidative stress components that can kill bacteria within the cell (Janeway et al., 2001). To antagonize the collective production of these toxic products, cells were treated with the NADPH oxidase inhibitor diphenyleneiodonium chloride (DPI). While the overall levels of uptake were lower in DPI treated samples, the overall pattern demonstrating a lower uptake of immune serum opsonized MAP remains the same as with the addition of other inhibitors. It was noted that DPI-treated MAP controls result in a 35% decrease in the viability of the bacteria, while there was a 10% decrease in host cell viability in DPI-treated controls compared to DMSO vehicle treated samples, accounting for the decrease in overall uptake in the DPI-treated assays. All other controls demonstrate negligible changes in viability of either MAP or host cell controls during treatment with inhibitors or their respective vehicle controls (data not shown).

**Figure 5:**
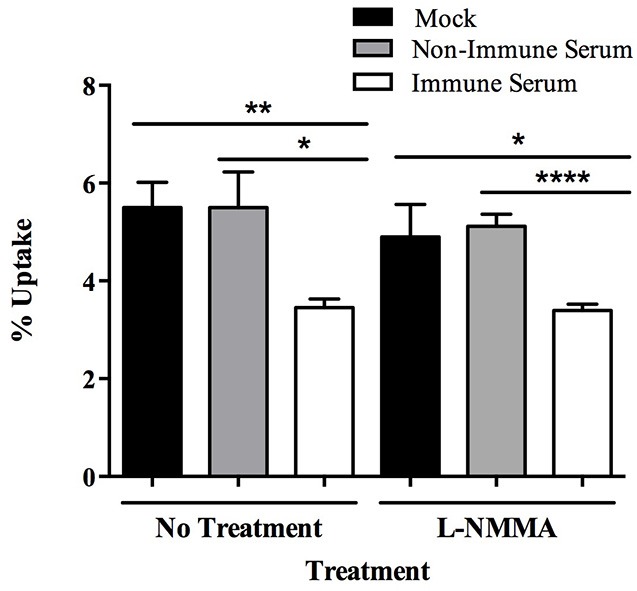
**Role of nitric oxide in the killing of immune serum opsonized MAP during BOMAC infection**. BOMAC cells were pre-incubated with the cellular mechanism inhibitor L-NMMA (250 μM) or water vehicle control in RPMI medium for 30 min prior to infection. Cells were infected with 10^7^ mock or serum opsonized milk-exposed MAP in RPMI in the presence of inhibitor and buffer alone. After 15 min infection, samples were washed, lysed, and quantified. Data shown represent mean ± SEM of two independent experiments each performed in triplicate (^*^p < 0.05, ^**^p < 0.01, ^****^p < 0.0001 as determined by Student's *t*-test).

In addition to the stimulation of an oxidative burst upon phagocytosis, host cells can mount a nitrosative burst, specifically by the production of nitric oxide, to aid in the destruction of ingested pathogens. To analyze the role of nitric oxide during phagocytosis of opsonized milk-exposed MAP, treatment was completed with the nitric oxide inhibitor *N*^*G*^-monomethyl-l-arginine (L-NMMA). Treatment with L-NMMA results in no change in uptake and intracellular bacterial load of immune serum opsonized MAP compared to control treated samples (Figure 5). There was no effect on viability of the MAP or cell populations upon addition of L-NMMA in controls (data not shown).

**Figure 6:**
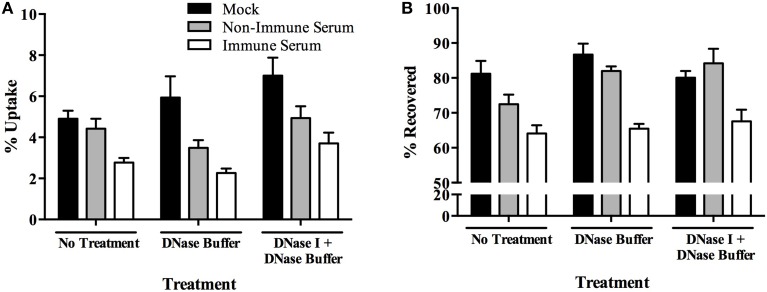
**Role of macrophage extracellular traps (METs) in the killing of immune serum opsonized MAP during BOMAC infection**. BOMAC cells were pre-incubated with DNase I/DNase buffer (100 U/ml) or DNase buffer alone in RPMI medium for 30 min prior to infection. Cells were infected with 10^7^ mock or serum opsonized milk-exposed MAP in RPMI in the presence of inhibitor and buffer alone. After 15 min, infected cells were washed, lysed, and intracellular bacteria **(A)** and bacteria-containing supernatant samples **(B)** were quantified. Data shown represent mean ± SEM of two independent experiments each performed in triplicate.

To identify if extracellular killing mechanisms were responsible for the rapid killing of immune serum opsonized MAP, we investigated the role of extracellular traps during our infection model. Neutrophil extracellular traps (NETs) have been described as a toxic tool employed upon infection with both gram-negative and gram-positive organisms (Brinkmann et al., 2004; Aulik et al., 2012). Upon exposure to stimuli, cells release a sticky DNA net into the extracellular environment which is characterized by the presence of histones and elastase enzymes. This phenomenon has recently been reported to be used by macrophages as well, resulting in macrophage extracellular traps (METs) in response to TNF-α (Mohanan et al., 2013) and a variety of pathogens and toxins (Aulik et al., 2012; Liu et al., 2014). To determine if METs were stimulated in response to infection with our bacterial populations, BOMAC cultures were treated with DNase prior to and during each uptake assay. DNase treatment identifies that the dissolution of DNA complexes outside of the cell does not alter the pattern of selective immune-serum opsonized MAP killing (Figure 6A), nor does it alleviate the lack of extracellular immune serum opsonized MAP during the assays (Figure 6B).

### Opsonization of infectious MAP phenotype in a murine model system

**Figure 7:**
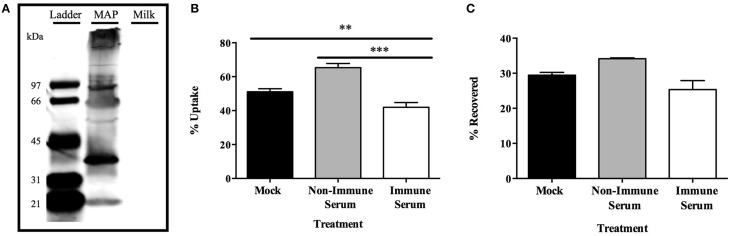
**Effect of opsonization on MAP survival in a murine model**. Serum was collected from mice after subcutaneous vaccination with protein preparations of the infectious phenotype of MAP over a 3 month timeframe. Equal amounts of proteins from either the infectious MAP phenotype or milk proteins only separated on an SDS-PAGE protein gel and Western blot was conducted using terminal serum as the primary probing antibody (1:5000), and probed with a goat α-mouse IR800 secondary antibody for visualization **(A)**. The infectious phenotype of MAP was opsonized with pre-bleed non-immune serum, terminal bleed immune serum, or PBS (mock) and used to infect RAW 264.7 macrophage cells for 15 min. Cell lysate **(B)** and supernatant **(C)** samples from each well were quantified for bacterial uptake and supernatant bacterial viability, respectively. Data shown represents the mean ± SEM of two experiments each performed in triplicate (^**^p < 0.01, ^***^p < 0.001 as determined by a Student's *t*-test).

The observation that bovine macrophages were able to more effectively eliminate the infectious phenotype of MAP after opsonization with immune serum in such a rapid manner has not previously been described prior to this study. To understand if the phenomenon was specific to the physiology of bovine macrophages or could be observed in other systems, we completed the opsonization of the infectious phenotype of MAP and infection in a murine model. We first produced antibodies against the infectious phenotype of MAP in C57BL/6 mice. Western blot analysis of infectious MAP lysates and milk protein alone confirm that collected mouse serum contained antibodies which recognize only MAP bacterial lysate proteins and not bovine milk proteins (Figure 7A). To identify the effect the collected mouse serum had on the interaction of opsonized infectious MAP with murine RAW 267.4 macrophages, we conducted similar 15 min macrophage infection assays as done with the bovine model (Figures 7B,C). Murine RAW 264.7 macrophages exhibit a significantly lower level of immune serum opsonized bacteria compared to non-immune serum and mock opsonized samples (Figure 7B). Additionally, the decrease in bacterial uptake by murine macrophages is not an effect of neutralization, as the lack of intracellular MAP in the uptake assays are not recovered from the supernatants of each culture (Figure 7C). These data validate that the pattern of rapid host cell mediated killing is consistent between both the bovine and murine *in vitro* model systems, and provides further support that the infectious phenotype of MAP serves as an important and relevant bacterial phenotype for studies involving opsonization, and the resulting rapid phagocyte killing, as a means of preventing infection in multiple species models.

## Discussion

At present, the most effective preventative measures against Johne's disease are strict calf management practices so as to avoid the exposure of neonates and young animals to colostrum, milk, feed, and soil contaminated with MAP. The currently approved vaccine protocol involves administration of the bacterin within a strict window between 0 and 30 days of age. Unfortunately, the vaccine can be ineffective at stopping the spread of the disease through a herd and results in variable protection against disease depending on the immune status, age, infection status, and other unknown factors about the animal. Treatment options are difficult and not generally encouraged as they include antibiotic regimens which are expensive, are required for the duration of the animal's life, and are often unable to fully eliminate the infection. Most often, infected animals are culled in order to prevent the transmission of MAP to the rest of the herd. Novel vaccination strategies would allow for more successful methods of preventing infection among young animals, thus slowing the cycle of transmission and incidence of Johne's disease within herds.

The delivery of antibodies in colostrum and milk is an important mechanism which passively transfers protective IgA and IgG to neonatal and suckling animals that are born with undeveloped and immature immune systems. The intestinal physiology of neonatal calves is quite unique within the first 24 h of life as the animal needs to take up as much protective immunoglobulin as it can to establish a protective immune system. Neonatal animals express increased levels of the neonatal Fc receptor (FcRn) in various tissues including the lung (Mayer et al., 2004), and intestine (Kuo et al., 2010). These receptors allow for the maximum level of absorption of maternal IgG molecules into circulation for the establishment of immunity and to provide local protection against pathogen exposure early in life. Meanwhile, IgA molecules are utilized as neutralizing protection against pathogens the young animal may encounter prior to the establishment of a complete immune system. The passive transfer of antibodies via milk from experimentally vaccinated dams can result in protection against commonly encountered farm pathogens (Reiter and Brock, 1975; Saif et al., 1983; Perryman et al., 1999). In this study we aimed to identify whether the shift to an infectious bacterial phenotype in milk (Patel et al., 2006), its direct transmission to young animals (Taylor et al., 1981; Sweeney et al., 1992), and the natural passage of maternally-derived antibodies would be useful as a protective mechanism against an intracellular pathogen such as MAP.

Our initial results suggest that antibodies do not play a neutralizing role during initial epithelial cell infection as there was no significant change in the invasion ability between opsonized and mock treated MAP after 1 h of infection. Alternatively, rather than protecting the epithelial barrier from infection, opsonization of the infectious MAP phenotype resulted in a significantly increased rate of trafficking of MAP across an intact polarized MDBK epithelial cell transwell culture. Epithelial cells, limited in their protective abilities, may be transporting opsonized MAP to a location where they are more likely to be appropriately dealt with by professional immune cells in the mucosal tissue for a more successful and positive outcome for the host. As MAP is an intracellular pathogen with an arsenal of mechanisms used to avoid host cell-mediated killing mechanisms, we asked whether the enhanced translocation across the epithelial cell layer was advantageous to the uptake by and survival within host phagocytes, or whether it resulted in a protective effect by triggering enhanced killing of the pathogen. Astoundingly, upon opsonization of MAP expressing the milk-induced infectious phenotype, the effect of opsonization changed from one of enhanced uptake (Figure 3A) to one that resulted in the immediately killing of the immune serum opsonized bacteria (Figure 3B). This effect was solely a result of the phenotype and antibody recognition, as preliminary experiments ruled out reagent and protocol influence. The decrease in uptake of immune serum opsonized MAP by macrophages was not an effect of neutralizing IgA molecules, as we would have expected to see a higher amount of unbound and non-phagocytized but viable MAP in the supernatants to make up for the decrease in intracellular MAP in each respective sample (Figures 3B,C). We predict that the dramatic change in the outcome of opsonized samples and the impact on MAP viability are an effect of the specific milk-induced infectious bacterial phenotype and the antibody recognition of serum from clinically infected cattle. The more effective match between surface exposed biomarkers and the deposition of antibodies during opsonization may be a key factor in the development of a more specific, and here shown as toxic, response by macrophages upon infection with immune serum opsonized, milk delivered MAP. A limitation of our work is that the system was only carried out using one type of bovine epithelial cells (MDBK) and the results with other epithelia cell type (such as bovine Mac-T mammary cell) could be different.

As we observed a rapid bacterial killing effect triggered by immune serum opsonization of MAP, we focused on identifying the specific innate immune mechanism responsible for the bacterial killing in our model. Uptake of opsonized pathogens results in the fusion of the bacteria-containing phagosome with intracellular lysosomes containing bactericidal components. The inhibition of the host cell-initiated mechanisms including catalase, superoxide anions, and the functional NADPH oxidase complex, resulted in no change in the viability of immune serum opsonized MAP during the infection of bovine macrophages, indicating that the oxidative burst triggered during uptake or phagosome-lysosome fusion is not responsible for the rapid killing of the bacteria (Figure 4). The nitrosative burst within host phagocytes can also be used to eliminate intracellular pathogens, although mycobacteria are described to inhibit the recruitment of nitric oxide synthase to the phagosomal membrane during macrophage infection (Miller et al., 2004), and the level of nitric oxide produced during infection only results in minimal mycobactericidal activity against MAP (Zhao et al., 1997). Regardless of the role of nitric oxide during intracellular MAP infection, inducible nitric oxide has been described to play a role in the response to opsonized particles, as demonstrated by luminescence assays in human macrophages (Gross et al., 1998); though it has also been described that IgG-opsonized particles induce apoptosis in macrophages in an oxidative-dependent and nitric oxide-independent manner (Kim et al., 2003). We illustrate that the inhibition of nitric oxide with L-NMMA resulted in no change in the intracellular viability of opsonized MAP in our model, indicating that the nitrosative burst is not responsible for the killing of the immune serum opsonized MAP upon macrophage infection. From these studies, we pinpoint a variety of cellular mechanisms that are not responsible for the killing of opsonized MAP. Further investigation into additional mechanisms of cellular protection including Cu/Zn toxicity and the activation of antibody-dependent cell-mediated cytotoxicity and their role in killing of opsonized MAP will elucidate the potential novel mechanism used to provide a toxic outcome for MAP infection within our model.

As an extracellular mechanism for eliminating pathogens, extracellular traps offer an efficient method of protection against infection in a tissue. The milieu that composes extracellular traps, including histones, enzymes such as elastase and a variety of cellular proteases, catch trap disease causing organisms, resulting in their destruction and degradation. Our investigation shows that the addition of DNase, which has the ability to disrupt the DNA matrix formed in the traps, thus rendering them unable to trap bacteria, has no effect on the killing on immune serum opsonized MAP in our model system (Figure 6). Furthermore, the formation of NETs is dependent on the ROS produced by NADPH oxidase activity during infection (Fuchs et al., 2007). Though it is unknown if the same is true for METs, the resulting inhibition of NADPH oxidase with DPI in our study, which shows no change in the host-mediated killing of immune serum opsonized MAP, offers further evidence toward METs not being the responsible cellular mechanism for the specific host-mediated killing in our model.

It is important to address the current inability for protection to be conferred to a neonate from a dam that is currently infected with MAP and should, in theory, be providing protective antibodies to her suckling calf. There are a variety of physiological, molecular, and immunological reasons that may explain why calves aren't currently protected by the ingestion of milk from an infected cow. It is quite possible that MAP-contaminated milk does transfer both specific and non-specific antibodies to the young calf which confers a certain level of protection against MAP infection. Transferred antibodies may provide a level of protection which increases the infectious dose required for infection to be established, or it may simply allow for a slower progression of the disease as fewer bacteria are able to invade the epithelial lining in the beginning of infection. It is also clear that MAP and the closely related *M. avium* subspecies *hominissuis* readily change their phenotype based on the environment they are in or the host they are infecting, including milk (Patel et al., 2006), amoeba (Cirillo et al., 1997; Tenant and Bermudez, 2006), bovine macrophages (Bermudez et al., 2004; Patel et al., 2006), and intracellular passage between epithelial cells and macrophages in the intestinal tissue. The phenotype that a young animal is initially infected with, that which is expressed by the bacterium as it begins to replicate and spread throughout the host intestinal tissues, and the phenotype that occurs upon the transition to the clinical, inflammatory stage of the disease are potentially each characterized by their own particular set of antigens that are different from one another. Depending on the phenotype of the bacterium to which the pregnant cow produced antibodies which would be naturally passed in the milk, it is possible that complete or even partial protection may not be provided due to the mismatch between the dominant antigen epitopes of the bacterium and the maternal antibody recognition sites from her mounted humoral response. Overall, little is known about the protection that maternal antibodies delivered in colostrum and milk provide against MAP infection, if it exists at all; though it is clear that full protective passive immunity is not transferred as calves ultimately become infected with MAP from early life feedings. As seen with other pathogens (Reiter and Brock, 1975; Saif et al., 1983), it is a possibility that the most protective antibody titers against MAP would have to be induced by a vaccination with the relevant set of infectious MAP antigens during pregnancy in order for the production and adequate passage of protective antibodies to her calf.

To further understand the effect of passively transferred antibodies and the protective effect against MAP infection, animal models must be employed. A mouse model would serve as a simple *in vivo* model to understand how the opsonization of MAP affects the uptake of MAP by the intestinal enterocytes as well as intestinal Peyer's patches. Additionally, a mouse model could further elucidate the role of tissue macrophages as well as other intestinal innate immune cells and their respective roles in the survival and/or killing of antibody-opsonized MAP during infection. However, while mouse models would serve as more simplified animal models to further study the mechanisms described above, the neonatal calf would serve as the ideal animal model. The uptake of colostrum and milk immunoglobulins is dependent on the unique and specific physiology of the neonatal calf. Antibodies are able to more likely survive degradation due to the lack of digestive enzymes such as pepsin which are secreted in low amounts in the first hours of life (Thivend et al., 1980), and only in the intestinal epithelium of newborn calves are antibodies highly transported across the epithelial barrier which begins to mature, divide, and set the appropriate intestinal acidic pH as early as 12 h after birth (Stott et al., 1979; Kuo et al., 2010). Using a neonate model would appropriately test the protective ability of passive immunity and its effect in protecting against Johne's disease derived from MAP contaminated milk and colostrum sources.

In total, we demonstrate that bacterial incubation with serum containing opsonizing antibodies confers a protective effect against the initiation of infection of MAP in bovine and murine *in vitro* cell models. Opsonization of a relevant infectious phenotype that is present at the immediate time of passage from dam to calf results in greater translocation of bacteria across polarized epithelial cells. Furthermore, additional experiments demonstrate that rapid killing by macrophage-mediated bactericidal mechanisms is employed by specifically eliminating antibody-opsonized MAP. These data offer evidence toward a novel preventative strategy that takes advantage of the infectious phenotype that MAP expresses at the time of transfer from cow to calf in the milk. The passive transfer of anti-MAP antibodies and enhanced immunity in the early hours of the life of a calf may be able to prevent the initiation of disease in young animals, and thus stop infection and the development and transmission of clinical Johne's disease among herds. Ongoing studies are identifying the specific antigens in MAP associated with protection, and whether the same antibody response in present in the milk. The results of these studies can potentially help in the development of a mucosal vaccine.

### Conflict of interest statement

The authors declare that the research was conducted in the absence of any commercial or financial relationships that could be construed as a potential conflict of interest.
